# Effectiveness of Tralokinumab Across Atopic Dermatitis Phenotypes

**DOI:** 10.3390/jcm14062077

**Published:** 2025-03-18

**Authors:** Francesca Barei, Paolo Calzari, Elena Pezzolo, Maddalena Napolitano, Mariateresa Rossi, Mario Bruno Guanti, Francesca Caroppo, Anna Belloni Fortina, Cataldo Patruno, Anna Campanati, Tommaso Bianchelli, Giovanni Marco D’Agostino, Eustachio Nettis, Francesco Pugliese, Francesca di Vico, Ilaria Trave, Emanuele Cozzani, Luca Stingeni, Katharina Hansel, Matilde Dall’Olio, Laura Grigolato, Rosa Coppola, Vincenzo Panasiti, Martina Maurelli, Giampiero Girolomoni, Michela Ortoncelli, Simone Ribero, Angelo Valerio Marzano, Silvia Mariel Ferrucci

**Affiliations:** 1 Dermatology Unit, Fondazione IRCCS Ca’ Granda Ospedale Maggiore Policlinico, 20122 Milan, Italy; 2 Department of Pathophysiology and Transplantation, Università degli Studi di Milano, 20122 Milan, Italy; 3 Dermatology Unit, Ospedale San Bortolo, 36100 Vicenza, Italy; 4 Section of Dermatology, Department of Clinical Medicine and Surgery, University of Naples Federico II, 80138 Napoli, Italy; 5 Dermatology Unit, Spedali Civili Hospital, 25123 Brescia, Italy; 6 Struttura Complessa di Dermatologia, Azienda Ospedaliera Universitaria Policlinico di Modena, 41125 Modena, Italy; 7 Dermatology Unit, Department of Medicine (DIMED), University of Padua, 35122 Padua, Italy; 8 Pediatric Dermatology Regional Center, Department of Women and Children’s Health (SDB), University of Padua, 35122 Padua, Italy; 9 Dipartimento di Scienze della Salute, Università Magna Graecia di Catanzaro, 88100 Catanzaro, Italy; 10 Dermatology Clinic, Department of Clinical and Molecular Sciences, Polytechnic Marche University, 60121 Ancona, Italy; 11 Dermatology Unit, Istituto Nazionale di Riposo e Cura per Anziani, INRCA-IRCCS Hospital, 60121 Ancona, Italy; 12 Department of Precision and Regenerative Medicine and Ionian Area, University of Bari Aldo Moro, 70121 Bari, Italy; 13 Section of Dermatology, Department of Health Sciences (DISSAL), University of Genoa, IRCCS Ospedale Policlinico San Martino, 16132 Genoa, Italy; 14 Dermatology Section, Department of Medicine and Surgery, University of Perugia, 06123 Perugia, Italy; 15 Dipartimento di Scienze del Farmaco, Università degli Studi di Padova, 35122 Padova, Italy; 16 Operative Research Unit of Dermatology, Fondazione Policlinico Universitario Campus Bio-Medico, Via Alvaro del Portillo, 200, 00128 Roma, Italy; 17 Department of Medicine and Surgery, Università Campus Bio-Medico di Roma, Via Alvaro del Portillo, 21, 00128 Roma, Italy; 18 Section of Dermatology, Department of Medicine, University of Verona, 37129 Verona, Italy; 19 Dermatology Clinic, University of Turin, 10124 Turin, Italy

**Keywords:** atopic dermatitis, tralokinumab

## Abstract

**Background/Objectives**: Tralokinumab, a fully human monoclonal antibody targeting IL-13, has shown efficacy and safety in clinical trials and real-life studies for atopic dermatitis (AD). However, data on its effectiveness across AD phenotypes are limited. **Methods**: A multicentric study evaluated tralokinumab’s efficacy over 52 weeks in 416 severe AD patients. EASI (Eczema Area and Severity Index), P-NRS (Pruritus Numerical Rating Scale), DLQI (Dermatology Life Quality Index), and ADCT (Atopic Dermatitis Control Tool) were recorded up to 52 weeks of treatment. **Results**: The EASI, P-NRS, DLQI, and ADCT trends across phenotypes showed significant improvement in all phenotype subgroups. By week 16, classical and generalized lichenoid phenotypes showed the highest EASI improvements compared to the generalized inflammatory (75.0 vs. 45.5 [*p* < 0.001] and 79.3 vs. 45.5 [*p* < 0.001]), with most achieving EASI-75 (*p* < 0.001, χ^2^ = 25.96). By week 24, generalized lichenoid reached 100% EASI improvement, significantly outperforming other phenotypes. The highest EASI-75 rates were seen in classical, generalized lichenoid, and portrait/head and neck phenotypes (*p* = 0.016, χ^2^ = 13.85). No significant differences were observed at weeks 32, 40, or 52. **Conclusions**: Our results suggest that tralokinumab’s durability and tolerability are consistent across the various phenotypes. The classical and generalized lichenoid were the fastest phenotypes to improve. However, given the uneven distribution of phenotypes and the gradual reduction in patient numbers over time, larger prospective studies are essential to confirm the observed trends.

## 1. Introduction

Atopic dermatitis (AD) is a chronic inflammatory skin condition marked by severe itching and recurring eczematous lesions. It is commonly associated with generalized skin dryness, an early onset of symptoms—often within the first two years of life—and a personal or family history of atopic disorders such as asthma, allergic rhinitis, or AD itself [1]. Central to its pathophysiology is the overactivation of type 2 immune responses, which are mediated by key cytokines such as interleukin (IL)-4 and IL-13. IL-4 and IL-13, both signaling through the IL-4 receptor α (IL-4Rα), are crucial in driving the inflammatory cascade, promoting T helper 2 (Th2) cell differentiation, and impairing epidermal barrier integrity by downregulating filaggrin and other structural proteins. These cytokines also enhance the production of IgE and contribute to eosinophilic inflammation [1]. Given the role of these cytokines, therapeutic strategies targeting IL-4 and IL-13 have emerged as promising approaches for AD management. AD can emerge in childhood or develop de novo in adulthood, exhibiting significant clinical variability. Unlike the classic flexural dermatitis often seen in children, adult-onset AD may present with distinct patterns. Diagnosing AD in adults can be challenging, as traditional diagnostic criteria were primarily designed for pediatric cases. Consequently, adult-onset AD is frequently a diagnosis of exclusion, particularly in new cases. With the exception of nummular eczema [2], it remains unclear whether different clinical phenotypes are associated with distinct cytokine expression patterns that could influence the response to biologics or JAK inhibitors. Despite its heterogeneity, AD is still regarded as a single disease and typically managed with a generalized treatment approach, highlighting the need for more tailored prevention and therapeutic strategies [3,4].

Tralokinumab is a fully human monoclonal antibody that specifically targets interleukin-13 (IL-13), a key cytokine involved in the inflammatory processes of AD. By neutralizing IL-13, tralokinumab helps reduce inflammation, improves skin barrier function, and alleviates symptoms such as itching and eczematous lesions. Approved for the treatment of moderate-to-severe AD in adults, tralokinumab has demonstrated efficacy and safety both in clinical trials and real-life studies [5,6,7,8,9,10,11,12]. Its dosing regimen involves an initial loading dose of 600 mg followed by maintenance dosing of 300 mg every two weeks.

Currently, limited data are available on the effectiveness of tralokinumab across different AD phenotypes, highlighting the need for further research to better understand its therapeutic potential in diverse patient populations. The only report is that of Pezzolo et al.’s [12] report on tralokinumab effectiveness in prurigo nodularis-like atopic dermatitis. To address this, a retrospective multicentric study evaluated tralokinumab drug survival and effectiveness in severe AD patients by phenotype.

## 2. Materials and Methods

### 2.1. Patients and Data Collection

The study enrolled 416 adult (≥18-year-old) patients with severe AD (defined as an Eczema Area and Severity Index [EASI] score ≥ 21.1) receiving tralokinumab across fifteen tertiary centers in Italy. Inclusion criteria consisted of adult patients who had been receiving tralokinumab treatment for at least 16 weeks and had a phenotype identifiable within the classification by Salvador et al. Patients with less than 16 weeks of therapy and those with phenotypes that were numerically underrepresented or not identifiable with one of Salvador et al. were excluded. Patients received an induction dose of 600 mg at baseline, followed by 300 mg every two weeks. The first patient initiated tralokinumab treatment in July 2021, with data lock completed in March 2024. Data were considered up to 52 weeks of observation from July 2021 Baseline data included sex, age, atopic and other comorbidities, predominant clinical AD phenotype [13], intrinsic vs. extrinsic patterns, atopic family history (FH), AD onset age (childhood or adolescence onset vs. adult-onset [≥18-year-old], and previous treatment. Clinimetric scores such as EASI and Patient Reported Outcomes (PROs) such as the Pruritus Numerical Rating Scale (P-NRS), Dermatology Life Quality Index (DLQI), and Atopic Dermatitis Control Tool (ADCT) were recorded. Given the retrospective nature of the study and variable follow-up intervals, data were collected at as many follow-up points as possible and aggregated every 8 weeks. Data at 8 weeks were not shown due to a small sample size. ChatGPT version 16 May 2024 (OpenAI, San Francisco, CA, USA) was used for grammar and sentence structure checker. 

### 2.2. AD Phenotypes

Phenotypes were classified according to Salvador et al. [13] (Table 1), as it is widely used on a regular basis by the authors of this study. The “classical” phenotype is the most common and is characterized by lichenified or exudative eczemas primarily affecting the head, neck, hands, and flexural areas. The “generalized lichenoid” phenotype presents with widespread lichenification, xerosis, and crusting, along with distinct signs of chronicity that involve both flexural and generalized body surfaces. The “generalized inflammatory” phenotype is marked by acute, exudative, and crusted eczematous lesions, sometimes accompanied by profuse scaling. The “nummular eczema (NE)-like” phenotype is characterized by round, inflamed lesions that primarily involve the lower limbs. The “prurigo nodularis (PN)-like” phenotype features intensely pruritic papules or nodules, typically located on the trunk and limbs. Finally, the “portrait and head and neck phenotype” involves eczema localized to the head and neck, which may extend to the chest and back, and might be associated with environmental sensitizations.

### 2.3. Statistical Analyses

Quantitative variables are summarized as mean ± standard deviation or median (Q1–Q3), depending on distribution, while categorical variables are reported as counts and frequencies. Chi-squared test or Kruskal–Wallis test (Bonferroni-corrected) were used as appropriate to assess a potential statistically significant difference among population groups. Wilcoxon test was used to assess potential differences from baseline data for each phenotype. DS was analyzed using unadjusted Kaplan–Meier survival curves to estimate the risk and time to discontinuation. Only the first cycle of treatment was considered, with patients who temporarily discontinued treatment for > 8 weeks considered as having discontinued treatment. Patients were considered to be ‘censored’ if, at data lock, they were still being treated with tralokinumab, lost to follow-up, or discontinued for non-drug-related causes [14]. We defined an ‘event’ as discontinuation due to primary or secondary inefficacy or adverse events. In our study, to calculate the sample size, the power*G software version 3.1 was used. Considering a factorial ANOVA test, with a significance level of 0.05 and 80% power, and assuming an effect size of 0.5, we determined that a minimum total sample size of 58 patients was necessary. Statistical analyses were two-tailed with a significance set at *p* < 0.05. Statistical analyses were carried out using SPSS version 29.0 (IBM, Armonk, NY, USA).

## 3. Results

### 3.1. Baseline Clinical and Epidemiological Characteristic

**Figure 1:**
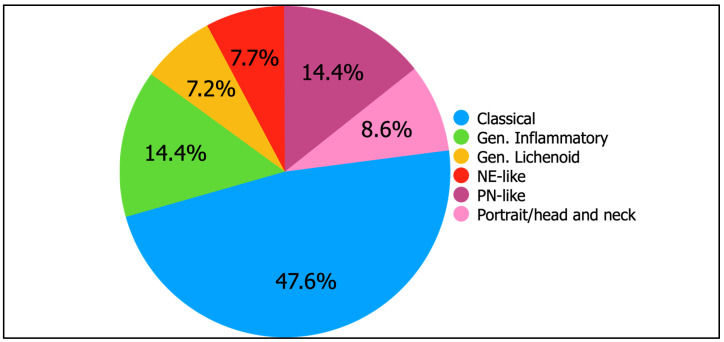
Distribution of phenotypes in the study population. Abbreviations: Gen., generalized; PN, prurigo nodularis; NE, nummular eczema.

The clinical and epidemiological characteristics at baseline, divided by phenotype, are represented in Table 2. The study included 416 patients, whose phenotypes were classical (198, 47.6%), generalized lichenoid (30, 7.2%), generalized inflammatory (60, 14.4%), nummular eczema (NE)-like (32, 7.7%), prurigo nodularis (PN)-like (60, 14.4%), and portrait/head and neck (36, 8.6%) [Figure 1]. Patients were all adults, with most of them (61.8%) having a childhood or adolescence onset. At baseline, some significant differences were found. Sex was almost equally distributed in the classical, generalized inflammatory and generalized lichenoid, while female sex was predominant in the PN-like and male sex was predominant in the NE-like and portrait/head and neck (*p* = 0.006, χ^2^ = 16.24). Childhood/adolescence-onset was common in classical, generalized, and portrait phenotypes, while adult onset predominated in NE-like and PN-like (*p* < 0.001, χ^2^ = 84.60). Extrinsic patterns were more frequent in classical, generalized, and portrait/head and neck phenotypes, while intrinsic patterns were common in NE-like and PN-like (*p* = 0.006, χ^2^ = 16.14). Most patients had at least one atopic comorbidity, except for PN-like patients (*p* < 0.001, χ^2^ = 22.68). The family history of AD was lower than 50% except for the generalized inflammatory (*p* < 0.001, χ^2^ = 38.63). The following differences were found for the baseline age: classical and NE-like (*p* = 0.001), classical and generalized lichenoid (*p* < 0.001), classical and generalized inflammatory (*p* < 0.001), classical and PN-like (*p* < 0.001), and portrait/head and neck and PN-like (*p* < 0.001).

### 3.2. Eczema Area and Severity Index

**Figure 2:**
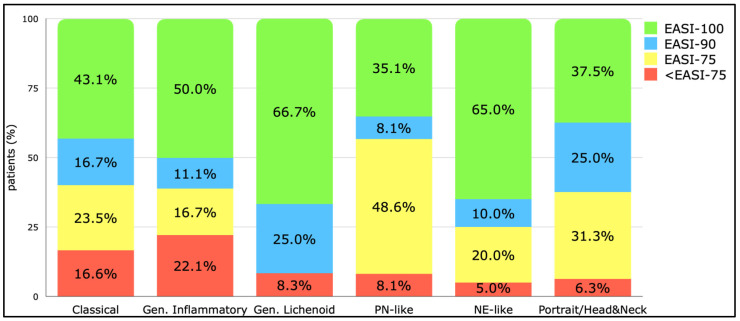
Endpoint of EASI-75, EASI-90, and EASI-100 at week 52 among the different phenotypes. Abbreviations: Gen., generalized; PN, prurigo nodularis; NE, nummular eczema.

The EASI trends across phenotypes are summarized in Table 3, showing significant improvement (*p* < 0.001) in all phenotype subgroups. At week 16, the following significant differences were observed when considering the EASI percentage improvement: classical vs. generalized inflammatory (75.0 vs. 45.5; *p* < 0.001) and generalized lichenoid vs. generalized inflammatory (79.3 vs. 45.5; *p* < 0.001). At week 24, significant differences included generalized lichenoid vs. classical (100.0 vs. 80.0; *p* = 0.025), generalized lichenoid vs. generalized inflammatory (100.0 vs. 75.5; *p* = 0.002), generalized lichenoid vs. NE-like (100.0 vs. 70.8; *p* = 0.007), and generalized lichenoid vs. PN-like (100.0 vs. 75.0; *p* = 0.031). By week 16, most patients with classical and generalized lichenoid phenotypes had achieved EASI-75 compared to other phenotypes (*p* < 0.001, χ^2^ = 25.96). By week 24, the highest percentages of EASI-75 achievement were seen in classical, generalized lichenoid, and portrait/head and neck phenotypes (*p* = 0.016, χ^2^ = 13.85). No significant differences were found at weeks 32, 40, or 52 [Figure 2].

### 3.3. Pruritus Numerical Rating Scale

The P-NRS trends across phenotypes are detailed in Table 4, demonstrating significant improvement (*p* < 0.001) in all phenotype subgroups. At week 16, the following significant differences in *p*-NRS percentage improvement were observed: generalized lichenoid vs. classical (80.0% vs. 55.6%; *p* = 0.035) and generalized lichenoid vs. generalized inflammatory (80.0% vs. 40.0%; *p* < 0.001). By week 24, a significant difference emerged between generalized lichenoid and NE-like phenotypes (100.0% vs. 50.0%; *p* = 0.009). When considering the endpoint P-NRS 0/1, a significant difference was found at week 16 (*p* = 0.002, χ^2^ = 19.04) and week 24 (*p* = 0.002, χ^2^ = 13.30), with the generalized lichenoid having the greatest percentage. No significant differences were detected at weeks 32, 40, or 52.

### 3.4. Dermatology Life Quality Index

The DLQI trends across phenotypes are detailed in Table 5, demonstrating significant improvement (*p* < 0.001) in all phenotype subgroups. At week 16 and 24, a significant difference in DLQI percentage improvement between generalized lichenoid and NE-like was found (W16: 66.7 vs. 40.0; *p* = 0.046; W24: 100.0 vs. 70.2; *p* = 0.032).

### 3.5. Atopic Dermatitis Control Tool

The ADCT trends across phenotypes are detailed in Table 6, demonstrating significant improvement (*p* < 0.001) in all phenotype subgroups. No significant differences were found among the phenotypes at any timepoint.

### 3.6. Safety and Drug Survival

The overall drug survival rate at week 52 was observed to be 83.2%, reflecting a high level of adherence and tolerability to the treatment over the one-year period. By week 52, five patients were lost to follow-up (two after the 16-week visit, two after the 24-week visit, and one after the 32-week visit) as they did not attend their scheduled follow-up appointments. A total of 53 cumulative discontinuations were recorded during the study: 16 by week 16, 33 by week 24, 41 by week 32, 42 by week 40, and 53 by week 52. Most of these events were attributed to inefficacy (44 cases, accounting for 83.0% of discontinuations). Other reasons for treatment cessation included psoriasiform reactions (five cases, 9.3%), ocular adverse events (three cases, 6.6%), and a single instance of inflammatory polyenthesitis (one case, 1.9%). Notably, when comparing the different phenotypes of atopic dermatitis, no statistically significant differences were observed in drug survival rates (*p* = 0.351; log-rank = 5.560).

## 4. Discussion

The EASI, P-NRS, DLQI, and ADCT trends across phenotypes showed significant improvement from baseline in all phenotype subgroups. The findings of this study suggest that tralokinumab offers consistent effectiveness across the various clinical phenotypes of AD. In clinical practice, AD presents with significant heterogeneity. These findings are particularly reassuring for clinicians, as they highlight tralokinumab’s effectiveness across various AD phenotypes, reinforcing its potential as a versatile therapeutic option even in non-classical cases.

Regarding potential differences between phenotypes, variations were observed at weeks 16 and 24. Specifically, in terms of EASI and P-NRS, the generalized inflammatory phenotype showed the slowest rate of improvement and had the lowest percentage of patients achieving EASI-75 by week 52. In contrast, the classical and generalized lichenoid phenotypes exhibited the greatest improvement in EASI and P-NRS during the first 24 weeks. However, it is important to note that the phenotypes are represented in different proportions, with the classical phenotype being the most prevalent. Speculation about the ADCT score is particularly challenging due to the high proportion of missing data compared to the other scores. The generalized lichenoid phenotype showed one of the fastest rates of improvement. This result might be linked to IL-13’s involvement in processes such as increased collagen deposition and fibrotic tissue remodeling, which are key contributors to the characteristic clinical features of this phenotype [15,16].

No significant differences in the clinimetric score were detected at weeks 32, 40, or 52. At 12 months, no notable differences in the treatment outcomes were detected among the phenotypes. Similarly, no statistically significant differences were observed in drug survival rates among the phenotypes. These results underscore the effectiveness of the drug across different phenotypes, providing consistent long-term outcomes. It should, however, be noted that the smaller sample size of some phenotypes may have impacted the statistical power of the analysis.

This is the first study aimed at evaluating the efficacy of tralokinumab across different clinical phenotypes of AD, and comparisons with other studies are limited. The effectiveness results of tralokinumab in the PN-like phenotype are consistent with the findings of Pezzolo et al. [12], supporting the growing evidence of its targeted efficacy. In prior research conducted by the group of Ferrucci, the efficacy of dupilumab was demonstrated across various phenotypes of atopic dermatitis (AD), highlighting that phenotypes such as the prurigo-like and NE-like exhibited slower clinical improvement compared to others [17]. Our findings align with these observations, showing that the NE-like phenotype demonstrates a slower progress during the initial months of therapy. However, despite the slower initial response, it achieves a remarkable median improvement of 100.0% in both EASI and P-NRS scores by week 52 of treatment. This significant improvement could potentially be explained by the higher levels of IL-13 observed in intrinsic AD when compared to extrinsic AD, as intrinsic AD was found to be more prevalent in patients with the NE-like phenotype [18,19,20]. Elevated IL-13 levels may play a critical role in the therapeutic response over time.

In summary, tralokinumab’s effectiveness is consistent across the various phenotypes in the long-term. Although these findings are promising, it is important to acknowledge the study’s limitations, including its retrospective design and the unequal distribution of phenotypes within the study population, which is an inherent bias, as the classic phenotype is the most prevalent. Also, it must be underlined that the progressive decline in patient numbers over time, which could have impacted the statistical power of the analysis, even though the proportional representation of phenotypes remained consistent throughout the study. Moreover, currently no direct correlation has been established between clinical phenotypes and endotype/cytokine profiles. As a result, no biologically grounded speculation can be made regarding potential differences between phenotypes.

This is the first study that aimed to evaluate the efficacy of tralokinumab in different clinical phenotypes of AD. Further research is necessary to validate and strengthen the robustness and reliability of these findings and enhance their clinical applicability. Larger prospective studies with a more balanced distribution of phenotypes are crucial to validate the observed trends and gain a deeper understanding of tralokinumab’s role in treating the diverse phenotypic expressions of AD.

## Figures and Tables

**Table 1:** Atopic dermatitis phenotype classification according to Salvador et al.

**Classical**	The most common phenotype, characterized by lichenified or exudative eczemas primarily affecting the head, neck, hands, and flexural areas.
**Generalized lichenoid**	This phenotype presents with widespread lichenification, xerosis, and crusting, along with distinct signs of chronicity that involve both flexural and generalized body surfaces.
**Generalized inflammatory**	This phenotype is marked by acute, exudative, and crusted eczematous lesions, sometimes accompanied by profuse scaling.
**Nummular eczema-like**	This phenotype is characterized by round, inflamed lesions that primarily involve the lower limbs.
**Prurigo nodularis-like**	This phenotype features intensely pruritic papules or nodules, typically located on the trunk and limbs.
**Portrait/head and neck**	These phenotypes involve eczema localized to the head and neck, which may extend to the chest and back, and might be associated with environmental sensitizations.

**Table 2:** Baseline characteristics among the phenotype groups.

Characteristic	Classical	Gen. Inflammatory	Gen. Lichenoid	PN-like	NE-like	Portrait/Head and Neck
**Patients**, *n* (%)	198 (47.6)	60 (14.4)	30 (7.2)	60 (14.4)	32 (7.7)	36 (8.6)
**Sex**, *n* (%)						
- Female	106 (53.5)	29 (48.3)	14 (46.7)	36 (60.0)	7 (21.9)	13 (36.1)
- Male	92 (46.5)	31 (51.7)	16 (53.3)	24 (40.0)	25 (78.1)	23 (63.9)
**Onset AD**, *n* (%)						
- Childhood/adolescence-onset	151 (76.3)	37 (61.7)	14 (46.7)	11 (18.3)	10 (31.3)	23 (63.9)
- Adult-onset	41 (20.7)	22 (36.7)	16 (53.3)	49 (81.7)	22 (68.8)	11 (30.6)
**Baseline age,** median (Q1–Q3)	31.0 (23.8–46.3)	45.5 (30.3–76.0)	50.5 (32.0–64.8)	64.0 (52.0–74.0)	51.5 (40.3–66.8)	43.5 (27.3–53.0)
**Atopic comorbidities**, *n* (%)	136 (68.7)	46 (76.7)	24 (80.0)	25 (41.7)	20 (62.5)	25 (69.4)
**Atopic comorbidities**, *n* (%)						
- Rhinitis	90 (45.5)	29 (48.3)	19 (63.3)	10 (16.7)	14 (43.8)	18 (50.0)
- Conjunctivitis	80 (40.4)	21 (35.0)	17 (56.7)	9 (15.0)	14 (43.8)	13 (36.1)
- Asthma	65 (32.8)	32 (53.3)	12 (40.0)	12 (20.0)	6 (18.8)	5 (13.9)
- Nasal polyposis	9 (4.5)	0 (0.0)	1 (3.3)	2 (3.3)	4 (12.5)	1 (2.8)
- Food allergy	30 (15.2)	1 (1.7)	3 (10.0)	4 (6.7)	4 (12.5)	5 (13.9)
- Drug allergy	14 (7.1)	8 (13.3)	4 (13.3)	6 (10.0)	5 (15.6)	5 (13.9)
**AD pattern**, *n* (%)						
- Intrinsic pattern	66 (33.3)	22 (36.7)	10 (33.3)	35 (58.3)	16 (50.0)	10 (27.8)
- Extrinsic pattern	130 (65.7)	38 (63.3)	20 (66.7)	25 (41.7)	14 (43.8)	23 (63.9)
**Positive FH for AD**, *n* (%)	66 (33.3)	35 (58.3)	15 (50.0)	7 (11.7)	5 (15.6)	8 (22.2)

Abbreviations: Gen., generalized; PN, prurigo nodularis; NE, nummular eczema; FH, family history.

**Table 3:** Assessment of eczema assessment severity index at different time points among the phenotype groups.

Phenotype	Week 0 *n* = 416	Week 16 *n* = 401	Week 24 *n* = 317	Week 32 *n* = 276	Week 40 *n* = 206	Week 52 *n* = 205
**Classical**	*n = 198/198*	*n = 191/191*	*n = 147/147*	*n = 136/136*	*n = 99/100*	*n = 102/102*
Score, median (Q1–Q3)	24.0 (18.0–26.0)	6.0 (2.0–10.0)	5.0 (2.0–15.5)	3.0 (1.0–6.0)	2.0 (0.0–6.0)	1.0 (0.0–5.0)
% improv., median (Q1–Q3)	/	75.0 (53.5–87.5)	80.0 (63.2–91.7)	85.7 (71.9–97.7)	90.6 (76.7–100.0)	95.3 (80.6–100.0)
EASI-75, *n* (%)	/	100 (52.4)	91 (61.9)	101 (74.3)	79 (79.8)	85 (83.3)
EASI-90, *n* (%)	/	39 (20.4)	40 (27.2)	50 (36.8)	51 (51.5)	61 (59.8)
EASI-100, *n* (%)	/	16 (8.4)	22 (15.0)	33 (24.3)	35 (35.4)	44 (43.1)
**Gen. inflammatory**	*n = 60/60*	*n = 59/59*	*n = 40/40*	*n = 30/30*	*n = 20/20*	*n = 18/18*
Score, median (Q1–Q3)	22.0 (17.0–26.0)	12.0 (6.0–18.0)	5.0 (2.0–15.5)	3.0 (0.0–7.3)	1.3 (0.0–7.3)	0.5 (0.0–5.3)
% improv. median (Q1–Q3)	/	45.5 (13.6–70.0)	75.5 (36.9–87.5)	85.0 (60.2–100.0)	93.3 (73.8–100.0)	97.7 (71.9–100.0)
EASI-75, *n* (%)	/	10 (16.9)	20 (50.0)	22 (73.3)	15 (75.0)	14 (77.8)
EASI-90, *n* (%)	/	3 (5.1)	9 (22.5)	12 (40.0)	14 (70.0)	11 (61.1)
EASI-100, *n* (%)	/	0 (0.0)	6 (15.0)	9 (30.0)	9 (45.0)	9 (50.0)
**Gen. lichenoid**	*n = 30/30*	*n = 29/29*	*n = 27/27*	*n = 18/28*	*n = 13/13*	*n = 12/12*
Score, median (Q1–Q3)	26.0 (24.0–29.3)	6.0 (0.5–12.0)	0.0 (0.0–5.0)	0.0 (0.0–3.0)	0.0 (0.0–3.5)	0.0 (0.0–1.0)
% improv. median (Q1–Q3)	/	79.3 (57.3–97.9)	100.0 (80.0–100.0)	100.0 (89.0–100.0)	100.0 (86.6–100.0)	100.0 (96.0–100.0)
EASI-75, *n* (%)	/	15 (51.7)	23 (85.2)	15 (83.3)	11 (84.6)	11 (91.7)
EASI-90, *n* (%)	/	10 (34.5)	23 (85.2)	13 (72.2)	9 (69.2)	11 (91.7)
EASI-100, *n* (%)	/	7 (24.1)	16 (59.3)	10 (55.6)	8 (61.5)	8 (66.7)
**PN-like**	*n = 60/60*	*n = 59/59*	*n = 48/48*	*n = 45/45*	*n = 34/35*	*n = 37/37*
Score, median (Q1–Q3)	25.0 (17.8–30.0)	7.0 (3.0–16.0)	5.0 (3.0–9.0)	4.0 (1.0–7.0)	2.5 (0.0–5.0)	3.0 (0.0–5.5)
% improv., median (Q1–Q3)	/	65.6 (36.4–86.8)	75.0 (67.2–88.1)	84.8 (71.1–95.8)	88.4 (79.8–100.0)	88.0 (79.6–100.0)
EASI-75, *n* (%)	/	22 (37.3)	28 (58.3)	31 (68.9)	28 (82.4)	34 (91.9)
EASI-90, *n* (%)	/	11 (18.6)	11 (22.9)	16 (35.6)	16 (47.1)	16 (43.2)
EASI-100, *n* (%)	/	5 (8.5)	4 (8.3)	8 (17.8)	9 (26.5)	13 (35.1)
**NE-like**	*n = 32/32*	*n = 30/30*	*n = 25/25*	*n = 22/22*	*n = 21/21*	*n = 20/20*
Score, median (Q1–Q3)	24.0 (20.0–28.0)	8.5 (4.0–16.3)	7.0 (3.0–11.0)	4.5 (1.8–7.3)	1.0 (0.0–5.0)	0.0 (0.0–3.8)
% improv., median (Q1–Q3)	/	67.7 (39.1–83.3)	70.8 (60.7–89.4)	82.7 (71.3–93.9)	95.8 (82.6–100.0)	100.0 (87.2–100.0)
EASI-75, *n* (%)	/	11 (36.7)	11 (44.0)	16 (72.7)	18 (85.7)	19 (95.0)
EASI-90, *n* (%)	/	4 (13.3)	6 (24.0)	7 (31.8)	15 (71.4)	15 (75.0)
EASI-100, *n* (%)	/	3 (10.0)	2 (8.0)	2 (9.1)	10 (47.6)	13 (65.0)
**Portrait/Head and neck**	*n = 36/36*	*n = 33/33*	*n = 30/30*	*n = 25/26*	*n = 18/18*	*n = 16/16*
Score, median (Q1–Q3)	24.0 (16.0–24.0)	6.0 (2.2–12.2)	3.4 (0.0–6.0)	2.0 (0.0–4.5)	1.5 (0.0–5.3)	1.0 (0.0–4.4)
% improv., median (Q1–Q3)	/	66.7 (50.0–88.6)	82.8 (70.8–100.0)	91.7 (77.1–100.0)	92.7 (75.0–100.0)	93.5 (81.3–100.0)
EASI-75, *n* (%)	/	12 (36.4)	22 (73.3)	21 (84.0)	15 (83.3)	15 (93.8)
EASI-90, *n* (%)	/	7 (21.1)	11 (36.7)	14 (56.0)	11 (61.1)	10 (62.5)
EASI-100, *n* (%)	/	2 (6.1)	8 (26.7)	7 (28.0)	8 (44.4)	6 (37.5)

Abbreviations: Gen., generalized; PN, prurigo nodularis; NE, nummular eczema; improv., improvement; *n*, proportion of valid data.

**Table 4:** Assessment of Pruritus Numerical Rating Scale at different time points among the phenotype groups.

Phenotype	Week 0 *n* = 416	Week 16 *n* = 401	Week 24 *n* = 317	Week 32 *n* = 276	Week 40 *n* = 206	Week 52 *n* = 205
**Classical**	*n = 198/198*	*n = 185/191*	*n = 143/147*	*n = 134/136*	*n = 100/100*	*n = 102/102*
Score, median (Q1–Q3)	8.0 (7.0–9.3)	4.0 (2.0–6.0)	3.0 (1.0–5.0)	2.0 (0.0–3.0)	1.0 (0.0–3.0)	1.0 (0.0–3.0)
% improv., median (Q1–Q3)	/	55.6 (16.7–75.0)	62.5 (33.3–87.5)	76.4 (44.4–100.0)	87.5 (62.5–100.0)	88.9 (60.0–100.0)
P-NRS 0/1, n (%)	1 (0.5)	33 (17.8)	46 (32.2)	57 (42.5)	65 (65.0)	67 (65.7)
**Gen. inflammatory**	*n = 60/60*	*n = 59/59*	*n = 40/40*	*n = 30/30*	*n = 20/20*	*n = 18/18*
Score, median (Q1–Q3)	7.0 (6.0–8.0)	5.0 (2.0–6.0)	2.0 (1.0–5.0)	2.0 (0.0–3.5)	0.0 (0.0–2.8)	0.5 (0.0–1.0)
% improv., median (Q1–Q3)	/	40.0 (14.3–62.5)	60.0 (23.8–85.3)	66.7 (33.3–100.0)	100.0 (40.0–100.0)	95.0 (76.7–100.0)
P-NRS 0/1, n (%)	0 (0.0)	6 (10.2)	11 (27.5)	12 (40.0)	12 (60.0)	15 (83.3)
**Gen. lichenoid**	*n = 30/30*	*n = 29/29*	*n = 27/27*	*n = 18/30*	*n = 13/20*	*n = 12/12*
Score, median (Q1–Q3)	9.0 (7.8–10.0)	2.0 (0.0–4.5)	0.0 (0.0–3.0)	0.0 (0.0–1.5)	0.0 (0.0–4.0)	0.0 (0.0–1.0)
% improv., median (Q1–Q3)	/	80.0 (30.0–100.0)	100.0 (40.0–100.0)	100.0 (85.0–100.0)	100.0 (58.9–100.0)	100.0 (89.2–100.0)
P-NRS 0/1, n (%)	0 (0.0)	14 (48.3)	19 (70.4)	14 (77.8)	9 (69.2)	10 (83.3)
**PN-like**	*n = 60/60*	*n = 59/59*	*n = 46/48*	*n = 44/45*	*n = 35/35*	*n = 37/37*
Score, median (Q1–Q3)	9.0 (7.3–10.0)	4.0 (2.0–6.0)	3.0 (1.8–5.0)	2.0 (0.3–4.8)	0.0 (0.0–3.0)	1.0 (0.0–4.0)
% improv., median (Q1–Q3)	/	55.5 (25.0–71.4)	64.6 (40.0–81.4)	77.7 (50.0–97.5)	100.0 (70.0–100.0)	90.0 (50.0–100.0)
P-NRS 0/1, n (%)	0 (0.0)	11 (18.6)	11 (23.9)	20 (45.5)	23 (65.7)	22 (59.5)
**NE-like**	*n = 32/32*	*n = 30/30*	*n = 24/25*	*n = 22/22*	*n = 21/34*	*n = 20/20*
Score, median (Q1–Q3)	8.0 (7.0–8.0)	4.0 (2.0–6.3)	5.0 (1.0–5.8)	2.0 (0.8–4.0)	1.0 (0.0–2.5)	0.0 (0.0–2.0)
% improv., median (Q1–Q3)	/	50.0 (9.4–75.0)	50.0 (3.6–85.7)	71.4 (37.5–92.5)	87.5 (68.8–100.0)	100.0 (75.7–100.0)
P-NRS 0/1, n (%)	0 (0.0)	6 (20.0)	7 (29.2)	9 (40.9)	15 (71.4)	13 (65.0)
**Portrait/Head and neck**	*n = 36/36*	*n = 31/33*	*n = 29/30*	*n = 26/26*	*n = 17/18*	*n = 16/16*
Sore, median (Q1–Q3)	8.0 (6.0–10.0)	4.0 (2.0–6.0)	3.0 (0.0–7.0)	3.0 (0.0–5.3)	1.0 (0.0–6.0)	1.5 (0.0–5.0)
% improv., median (Q1–Q3)	/	55.6 (25.0–71.4)	57.1 (21.1–100.0)	52.8 (21.7–100.0)	88.9 (33.3–100.0)	72.8 (35.0–100.0)
P-NRS 0/1, n (%)	1 (2.8)	6 (19.4)	10 (34.5)	11 (42.3)	10 (58.8)	8 (50.0)

Abbreviations: Gen., generalized; PN, prurigo nodularis; NE, nummular eczema; improv., improvement; *n*, proportion of valid data.

**Table 5:** Assessment of Dermatology Life Quality Index at different time points among the phenotype groups.

Phenotype	Week 0 *n* = 416	Week 16 *n* = 401	Week 24 *n* = 317	Week 32 *n* = 276	Week 40 *n* = 206	Week 52 *n* = 205
**Classical**	*n = 198/198*	*n = 185/191*	*n = 141/147*	*n = 133/136*	*n = 100/100*	*n = 102/102*
Sore, median (Q1–Q3)	13.5 (7.0–19.0)	4.0 (2.0–8.0)	3.0 (1.0–7.0)	2.0 (0.0–4.5)	1.0 (0.0–4.0)	1.0 (1.0–3.0)
% improv., median (Q1–Q3)	/	60.0 (24.0–84.6)	71.0 (33.3–94.7)	81.5 (50.0–100.0)	83.3 (66.7–100.0)	90.5 (68.8–100.0)
**Gen. inflammatory**	*n = 59/60*	*n = 58/59*	*n = 40/40*	*n = 30/30*	*n = 20/20*	*n = 18/18*
Sore, median (Q1–Q3)	18.0 (16.0–20.0)	9.0 (3.0–15.0)	3.0 (2.0–9.5)	2.0 (0.0–3.0)	0.0 (0.0–2.0)	0.5 (0.0–2.0)
% improv., median (Q1–Q3)	/	45.0 (8.7–71.0)	73.1 (25.0–88.9)	85.7 (65.0–100.0)	100.0 (66.6–100.0)	97.6 (88.3–100.0)
**Gen. lichenoid**	*n = 30/30*	*n = 29/29*	*n = 27/27*	*n = 18/30*	*n = 13/20*	*n = 12/12*
Score, median (Q1–Q3)	11.0 (10.0–20.0)	5.0 (0.0–10.5)	0.0 (0.0–5.0)	0.0 (0.0–3.3)	0.0 (0.0–2.0)	0.0 (0.0–1.5)
% improv., median (Q1–Q3)	/	66.7 (40.0–100.0)	100.0 (75.0–100.0)	100.0 (75.0–100.0)	100.0 (90.7–100.0)	100.0 (94.0–100.0)
**PN-like**	*n = 59/60*	*n = 58/59*	*n = 46/48*	*n = 44/45*	*n = 35/35*	*n = 37/37*
Score, median (Q1–Q3)	18.0 (11.0–25.0)	7.0 (4.0–10.0)	3.0 (2.0–8.3)	2.0 (0.0–4.0)	1.0 (0.0–4.0)	0.0 (0.0–3.0)
% improv., median (Q1–Q3)	/	54.5 (29.3–73.2)	72.7 (51.0–85.5)	92.6 (72.0–100.0)	96.2 (80.0–100.0)	100.0 (77.4–100.0)
**NE-like**	*n = 30/32*	*n = 30/30*	*n = 24/25*	*n = 22/22*	*n = 21/22*	*n = 20/22*
Score, median (Q1–Q3)	16.0 (6.8–27.3)	7.5 (4.8–15.0)	5.0 (3.0.8.0)	3.0 (0.8–6.0)	2.0 (1.0–5.0)	1.0 (0.0–2.8)
% improv. median (Q1–Q3)	/	40.0 (0.0–55.2)	70.2 (31.0–80.0)	79.3 (50.0–93.3)	85.2 (39.3–95.9)	93.1 (80.4–100.0)
**Portrait/Head and neck**	*n = 36/36*	*n = 32/33*	*n = 29/30*	*n = 26/26*	*n = 17/18*	*n = 16/16*
Score, median (Q1–Q3)	15.0 (9.3–21.8)	5.5 (2.0–11.0)	3.0 (1.0–13.0)	3.5 (0.0–10.3)	0.0 (0.0–7.0)	1.0 (0.0–10.0)
% improv., median (Q1–Q3)	/	51.0 (21.3–81.3)	70.0 (13.9–93.5)	77.5 (28.5–100.0)	100.0 (58.3–100.0)	85.0 (58.3–100.0)

Abbreviations: Gen., generalized; PN, prurigo nodularis; NE, nummular eczema; improv., improvement.; *n*, proportion of valid data.

**Table 6:** Assessment of Atopic Dermatitis Control Tool at different time points among the phenotype groups.

Phenotype	Week 0 *n* = 416	Week 16 *n* = 401	Week 24 *n* = 317	Week 32 *n* = 276	Week 40 *n* = 206	Week 52 *n* = 205
**Classical**	*n = 149/198*	*n = 146/191*	*n = 117/147*	*n = 107/136*	*n = 89/100*	*n = 82/102*
Sore, median (Q1–Q3)	17.0 (12.0–21.0)	7.0 (4.0–12.0)	6.0 (3.0–10.0)	3.0 (1.0–7.0)	4.0 (1.0–7.5)	3.0 (0.0–6.0)
% improv., median (Q1–Q3)	/	46.5 (25.4–75.0)	63.4 (30.4–83.9)	78.9 (50.0–95.7)	72.7 (60.2–93.6)	83.3 (66.7–100.0)
**Gen. inflammatory**	*n = 48/60*	*n = 47/59*	*n = 28/40*	*n = 22/30*	*n = 14/20*	*n = 11/18*
Sore, median (Q1–Q3)	6.0 (6.0–11.8)	5.0 (2.0–7.0)	3.0 (2.0–5.0)	2.0 (1.8–5.0)	5.0 (0.8–8.0)	3.0 (1.0–6.0)
% improv., median (Q1–Q3)	/	35.4 (16.7–60.)	50.0 (30.2–66.7)	62.5 (48.8–72.2)	62.5 (59.1–80.0)	80.0 (62.5–88.9)
**Gen. lichenoid**	*n = 12/20*	*n = 11/29*	*n = 9/27*	*n = 7/30*	*n = 7/20*	*n = 5/12*
Score, median (Q1–Q3)	18.5 (9.5–21.5)	12.0 (5.0–14.0)	12.0 (3.0–17.5)	4.0 (1.0–12.0)	4.0 (0.0–7.0)	1.0 (0.0–10.0)
% improv., median (Q1–Q3)	/	29.4 (13.6–68.1)	29.4 (5.2–81.4)	81.8 (45.5–95.0)	81.8 (58.8–100.0)	95.5 (50.5–100.0)
**PN-like**	*n = 25/60*	*n = 35/59*	*n = 27/48*	*n = 26/45*	*n = 19/35*	*n = 20/32*
Score, median (Q1–Q3)	18.0 (12.0–22.0)	7.0 (4.0–10.0)	5.0 (3.0–11.0)	3.0 (0.8–7.5)	2.0 (0.0–10.0)	2.5 (0.0–6.8)
% improv., median (Q1–Q3)	/	59.8 (20.6–78.1)	73.6 (21.2–86.7)	86.4 (47.9–97.9)	90.6 (58.7–100.0)	91.7 (69.2–100.0)
**NE-like**	*n = 25/32*	*n = 24/30*	*n = 18/25*	*n = 16/22*	*n = 15/22*	*n = 13/22*
Score, median (Q1–Q3)	20.0 (13.5–22.0)	11.5 (8.0–15.8)	10.0 (6.0–15.3)	8.0 (2.3–10.8)	5.0 (1.0–8.0)	4.0 (1.0–7.0)
% improv. median (Q1–Q3)	/	27.9 (4.4–55.6)	50.0 (31.8–63.9)	56.6 (43.8–89.9)	75.0 (58.3–95.5)	82.6 (65.5–93.6)
**Portrait/Head and neck**	*n = 26/36*	*n = 25/33*	*n = 21/30*	*n = 18/26*	*n = 12/28*	*n = 16/16*
Score, median (Q1–Q3)	18.0 (11.8–20.3)	7.0 (3.5–13.5)	8.0 (3.0–16.5)	7.0 (2.8–12.0)	3.0 (2.0–9.0)	2.5 (0.8–10.3)
% improv., median (Q1–Q3)	/	36.4 (13.6–78.9)	37.5 (10.5–72.5)	65.0 (22.9–81.2)	78.9 (54.8–89.9)	80.0 (42.4–95.7)

Abbreviations: Gen., generalized; PN, prurigo nodularis; NE, nummular eczema; improv., improvement.; *n*, proportion of valid data.

## Data Availability

Data are unavailable due to privacy restrictions.

## Abbreviations

The following abbreviations are used in this manuscript: Gen., generalized; PN, prurigo nodularis; NE, nummular eczema; FH, family history; EASI, Eczema Area and Severity Index; DLQI, Dermatology Life Quality Index, P-NRS, Pruritus Numerical Rating Scale.
